# Quantitative and qualitative estimation of atherosclerotic plaque burden in vivo at 7T MRI using Gadospin F in comparison to en face preparation evaluated in ApoE KO mice

**DOI:** 10.1371/journal.pone.0180407

**Published:** 2017-08-03

**Authors:** Caroline Jung, Sabine Christiansen, Michael Gerhard Kaul, Eva Koziolek, Rudolph Reimer, Jörg Heeren, Gerhard Adam, Markus Heine, Harald Ittrich

**Affiliations:** 1 Department of Diagnostic and Interventional Radiology and Nuclear Medicine, University Medical Center Hamburg-Eppendorf, Hamburg, Germany; 2 Department of Nuclear Medicine, Berlin Experimental Radionuclide Imaging Center (BERIC), University Medical Center Charité, Berlin, Germany; 3 Heinrich-Pette-Institute, Leibniz Institute for Experimental Virology, Hamburg, Germany; 4 Department of Biochemistry and Molecular Cell Biology, University Medical Center Hamburg-Eppendorf, Hamburg, Germany; Nagoya University, JAPAN

## Abstract

**Background:**

The aim of the study was to quantify atherosclerotic plaque burden by volumetric assessment and T1 relaxivity measurement at 7T MRI using Gadospin F (GDF) in comparison to *en face* based measurements.

**Methods and results:**

9-weeks old ApoE^-/-^ (n = 5 for each group) and wildtype mice (n = 5) were set on high fat diet (HFD). Progression group received MRI at 9, 13, 17 and 21 weeks after HFD initiation. Regression group was reswitched to chow diet (CD) after 13 weeks HFD and monitored with MRI for 12 weeks. MRI was performed before and two hours after iv injection of GDF (100 μmol/kg) at 7T (Clinscan, Bruker) acquiring a 3D inversion recovery gradient echo sequence and T1 Mapping using Saturation Recovery sequences. Subsequently, aortas were prepared for *en face* analysis using confocal microscopy. Total plaque volume (TPV) and T1 relaxivity were estimated using ImageJ (V. 1.44p, NIH, USA).

2D and 3D *en face* analysis showed a strong and exponential increase of plaque burden over time, while plaque burden in regression group was less pronounced. Correspondent *in vivo* MRI measurements revealed a more linear increase of TPV and T1 relaxivity for regression group. A significant correlation was observed between 2D and 3D *en face* analysis (r = 0.79; p<0.001) as well as between 2D / 3D *en face* analysis and MRI (r = 0.79; p<0.001; r = 0.85; p<0.001) and delta R1 (r = 0.79; p<0.001; r = 0.69; p<0.01).

**Conclusion:**

GDF-enhanced *in vivo* MRI is a powerful non-invasive imaging technique in mice allowing for reliable estimation of atherosclerotic plaque burden, monitoring of disease progression and regression in preclinical studies.

## Introduction

Atherosclerosis is the underlying cause of life-threatening cardiovascular events such as myocardial infarction and stroke and represents the leading cause of morbidity and mortality [1]. Animal models have become increasingly important tools to address key mechanistic and therapeutic questions that cannot be answered from human studies [2]. Therapies aimed at reducing the risk factors for developing atherosclerosis and/or directly reducing the size of the plaque are being actively pursued, creating the need for more robust methods to determine plaque alterations in preclinical models of atherosclerosis [3,4].

Different methods, most of them *ex vivo*, have been used to assess the extent of atherosclerotic plaque burden. One standard method involves serial sectioning of the heart and the aortic root [5] and subsequent histopathologic analysis to score and measure atherosclerotic lesions. Another method employs Sudan IV staining to determine the extent of atherosclerosis affecting the intimal surface throughout the entire aorta [6], which is commonly known as the *en face* technique [7]. For *en face* preparation the aorta is dissected from the heart to the iliac bifurcation, opened longitudinally to expose the luminal side and stained with Sudan IV so that lipid-rich plaques can be identified and lesional surface areas surveyed. Even if this traditional histopathology analysis remains the gold-standard technique for the assessment of atherosclerotic plaque burden, above described methods are non-serial, labour-intensive, time consuming and often rely on subjective, qualitative measures [8]. Moreover, histological preparation and processing not only introduces size distortion during tissue dehydration, but may disrupt the integrity of the plaque itself and is usually confined to 2D analyses. Other *ex vivo* techniques which have been used to detect and quantify the plaque volume include high resolution MRI [9] and μCT [8]. Although those imaging techniques allow a detailed look into the plaque, they are still invasive and require extensive animal numbers. For that reason, there is an increasing demand for alternative methods such as non-invasive and serial imaging technologies to supplement or even substitute *ex vivo* imaging techniques or histopathology. This would allow performing longitudinal preclinical studies on the development of plaque burden, the progression of disease and the evaluation of therapy response.

Molecular MRI and in particular contrast-enhanced MRI using extracellular gadolinium (Gd)-based contrast agent like Gadofluorine M (GDM; Bayer Schering Pharma AG, Germany) have proven to be promising tools allowing for evaluation of plaque composition in animal models of atherosclerosis [10]. Almost all studies using GDM for plaque detection were performed with rabbits and 1.5 or 3T MRI [11–13]. For studies using mice, MR imaging was performed at 11.7 Tesla and cardiac triggering [14]. Due to the lack of commercial availability of GDM new contrast agent, like Gadospin F (GDF) needed to be established and evaluated to perform contrast-enhanced MRI in atherosclerotic mice models. GDF is an amphiphilic gadolinium-based contrast agent like GDM and chemical structure is similar. Several groups have theorized about how GDM accumulates within the plaques [13], showing that GDM binds to lipidic components of plaques via interactions with its hydrophobic tail [11] or showing that GDM does preferentially bind to collagenous (fibrous) material within the plaque [15].

The aim of our study was to quantify atherosclerotic plaque progression and regression *in vivo* by volumetric assessment and determination of T1 relaxivity at 7T MRI using GDF in comparison to conventional *en face* preparation method. In addition, confocal microscopy was validated as a 3D visualization technique for atherosclerotic plaques and 3D plaque volume estimation *ex vivo* based on *en face* preparation.

## Material and methods

### Contrast agent

GDF (Miltenyi Biotec GmbH, Bergisch Gladbach, Germany) is a derivate of Gd-DO3A and an amphiphilic, low-molecular weight (1.3 g/mol) gadolinium based contrast agent containing a cyclic Gadolinium chelate and a perfluorinated side chain. Due to the lipophilic character of the perfluorinated side chain GDF assembles as small aggregates or micelles in diluted solution. Although different molecular binding structures are discusses, a predominant accumulation within atherosclerotic plaques of the aortic wall can be observed [11–13,16–18]. GDF has an r1 relaxivity of 18mmol/L^-1^s^-1^ and r2 relaxivity of 31mmol/L^-1^s^-1^ in plasma (Gd-DTPA 4mmol/L^-1^s^-1^ and 5mmol/L^-1^s^-1^ respectively) and r1 relaxivity of 15mmol/L^-1^s^-1^ and r2 relaxivity of 20 mmol/L^-1^s^-1^ in water (Gd-DTPA 3mmol/L^-1^s^-1^ and 4mmol/L^-1^s^-1^ respectively) (1.5T and 37°C).

### Animals and nutrition

**Fig 1:**
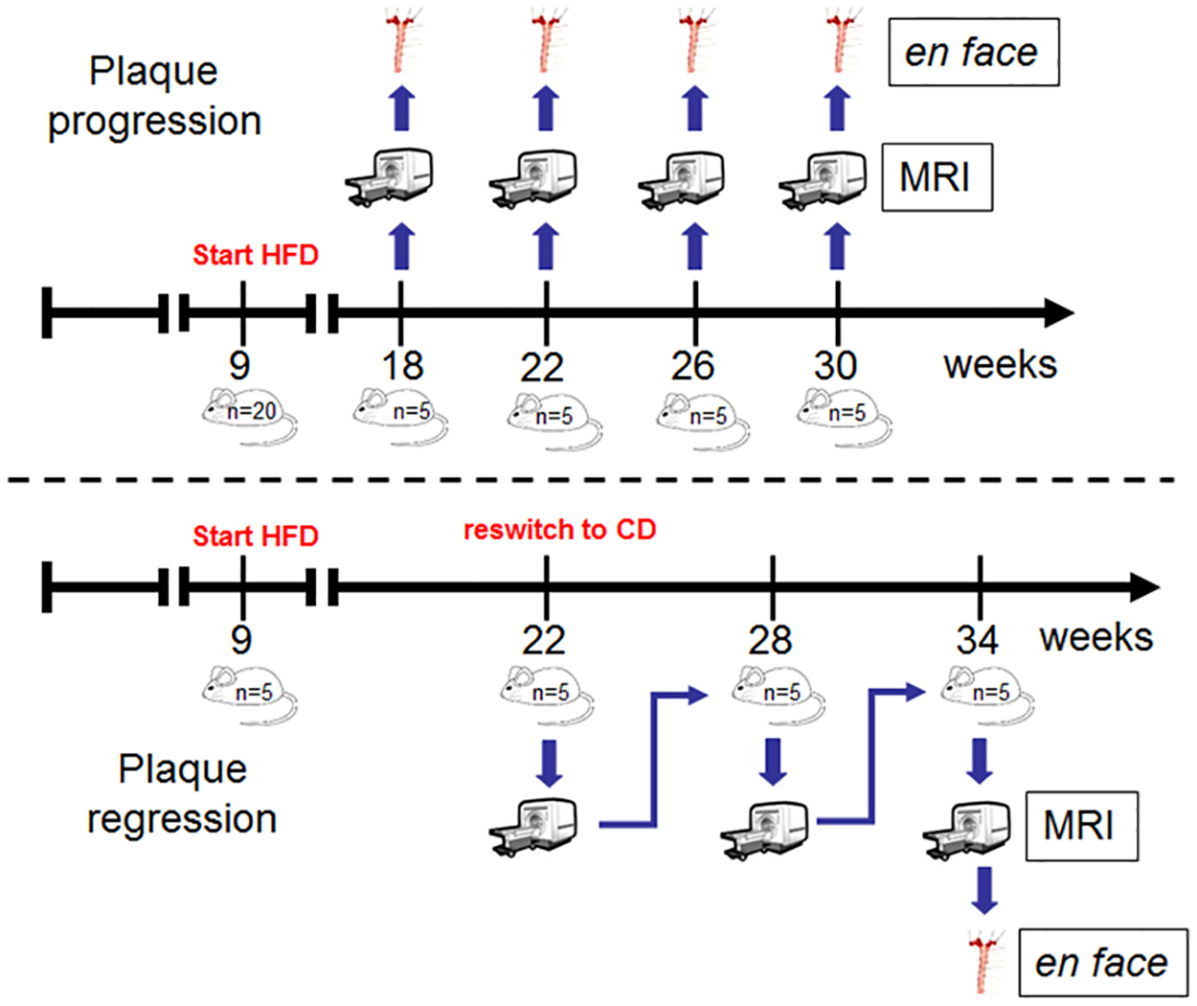
Study design. For atherosclerotic plaque progression analysis ApoE^-/-^ mice (n = 20; progression group) were set on high fat diet (HFD) at 9 weeks of age. Mice were than grouped (n = 5 for each time point) and MRI performed after 9, 13, 17 and 21 weeks on HFD. *En face* preparation was performed immediately after MRI. For atherosclerotic plaque regression analysis ApoE^-/-^ mice (n = 5, regressiony group) were also set on HFD at 9 weeks of age. First MRI examination was performed 13 weeks after starting the HFD. At this time point diet was switched back to chow diet (CD) and MRI analysis was repeated at the age of 28 and 34 weeks before *en face* preparation was done.

All B6.129P2-apoE^tm1Unc/J^ (C57BL/6J background) mice (ApoE^-/-^) were purchased from Charles River Laboratories / Jackson Laboratories (Bar Harbor, ME, USA) and were housed in the animal unit of the University Hospital Hamburg Eppendorf. C57BL/6J wildtype mice were used as controls. ApoE^-/-^ mice (n = 25) and wildtype mice (n = 5) were set on high fat diet (HFD; (ssniff^®^ EF R/M from TD88137 mod., ssniff GmbH) at 9 weeks of age. For progression analysis mice (progression group, n = 20) the HFD was continued for 9, 13, 17 and 21 (n = 5 each) weeks before MRI and histological analysis were performed. To monitor the regression of atherosclerotic plaque burden one mice group (regression group n = 5) were switched back to chow diet (CD) after 13 weeks HFD and CD was continued for 12 weeks (Fig 1). None of the utilized animals became ill or died prior to the experimental endpoint and none of them received medical treatment.

### Magnetic resonance imaging

*In vivo* MRI of the aortic vessel wall was performed in the progression group at 9, 13, 17 and 21 weeks and in the regression group at 13, 19 and 25 weeks after initiation of the HFD. MRI examinations were carried out using a 7 Tesla preclinical animal MRI scanner (Bruker Clinscan, Ettlingen, Germany). Animals were anesthetized with 1–2% isoflurane/O_2_ gas mixture with a flow rate of 600ml/min. The vital parameters were controlled by measuring the respiratory breathing cycle (SA Instruments, Stony Brook, NY, USA) and the body temperature was maintained at 37 degrees during examination. A 4-channel mouse surface coil with a coil diameter of 20mm for signal receiving and a rat body coil for RF-transmission with a coil diameter of 72mm was used (Bruker, Ettlingen, Germany). The scan protocol included the survey scan followed by a parasagittal turboflash sequence to cover the aortic arch (Table 1).

**Table 1:** MRI sequence parameters.

protocol	MR sequence	TR (ms)	TE (ms)	TI (ms)	NSA	matrix	FA	voxel (mm^3^)	time (min)
1	parasagittal tfl	2450	2,6	1450	10	320x320	20	0.09 x 0.09 x 0.7	00:25
2	transversal tfl3d	650	2	250	6	192x192	20	0.18 x 0.18 x 0.18	09:29
3	transversal tfl3d	650	2	250	10	192 x 108	20	0.18 x 0.18 x 0.22	09:00
4	transversal tfl3d	1500	2	***	2	256 x 144	20	0.14 x 0.14 x 0.22	27:30*

*** 1200ms, 500ms, 350ms, 200ms, 77ms;

*each 05:30min

Next, in transversal direction to the parasagittal slice orientation a high resolution sequence for plaque volumetry was planned. This 3D inversion recovery sequence covered the thoracic aorta and consisted of an isotropic voxel size of 0.18×0.18×0.18 mm^3^.

For T1 relaxation time mapping this sequence was followed a saturation recovery sequence with varying delay times of 77, 200, 350, 500, 1200 ms. To facilitate the examination of the relaxation time mapping and to achieve a better anatomical information a second inversion recovery sequence in the same slice orientation and with the same slice thickness was performed. No ECG or breath triggering was used. Images were obtained before and two hours after intravenous injection of GDF (100 μmol/kg).

### MR image analyses

MRI post processing analyses were performed offline with a custom made software plugin (qMapIt) (M.G. Kaul, ImageJ conference 2012) for image processing software (ImageJ, NIH, USA). The plugin was used for T1 analysis and for the alignment of the high resolution measurement with the T1 images to transfer the region of interest.

For assessment of the total plaque volume (TPV) region of interests (ROIs) were placed manually surrounding the atherosclerotic lesion. To optimize the display, all images were adjusted for image brightness and contrast. For plaque volume measurements single plaque ROIs of each slice were summed up and expressed in mm^3^. MR images were reviewed independently by two readers for assessment of interobserver agreement.

T1 relaxation time was achieved by a non-linear least square optimization using the Levenberg-Marquadt algorithm. Pixel by pixel the model function S(t) = S0 * [1—(1—B) * *exp* (-t / T1) —B * exp (-t / TR) / (1 –B * C * exp (-t / TR)] was fitted to measured data. S0 reflects the net magnetization in thermal equilibrium. B is the cosine of the saturation pulse and C of the excitation pulse. To restrict the degrees of freedom C was fixed to 1. The fit was performed in several passes with smoothing of B of the next neighbors assuming only slow varying B1 field changes.

ROIs of the plaque volumetry were transferred to the T1 maps after generating high resolution T1 maps with the same voxel sizes and in respect to shift of the field of view. ΔR1 was assessed by following equation: ΔR1 = R1_post_−R1_pre_ with R1 [s^-1^] = 1/T1[ms] × 1000, where R1_pre_ and R1_post_ represent the R1 relation rates before and after GDF injection.

### Histopathology and histomorphometry

Immediately after MRI measurements, each animal was sacrificed by an overdose of ketamine/xylazine (Ketamin Gräub, aniMedica GmbH, Senden-Bösensell, Germany; Rompun^®^, Bayer AG, Leverkusen, Germany). Before undergoing a whole-body cardiac perfusion with 4% paraformaldehyde (PFA), blood samples were taken from the left ventricle for plasma lipid analysis. Subsequently, the thoracic and abdominal aorta was isolated from all branches, excised and fixed for at least 24hours before flat-mounting on wax boards and staining with Sudan IV using previously published methods [19].

For 2D *en face* analysis photomicrographs of the prepared aortas were taken with a Nikon Coolpix digital camera (Nikon, Düsseldorf, Germany) mounted on a light microscope (Carl Zeiss, Jena, Germany). Images were captured under identical lighting, microscope, camera and PC conditions. Images were saved in tiff-format and were transferred to a PC for plaque area estimation using ImageJ. Compartment segmentation was performed by manually delineation of the atherosclerotic lesion.

The confocal laser scanning microscopy was done with a C2+ scanning head (Nikon GmbH, Düsseldorf, Germany) attached to a Nikon AZ100 multizoom microscope equipped with a 1x apochromatic objective and a motorized stage (Prior scientific, Cambridge, UK). 3D *en face* plaque volume was estimated by a semiautomatic tool for segmentation using NIS-Elements viewer (Nikon GmbH, Düsseldorf, Germany).

### Plasma lipids analyses

Blood was collected from the left ventricle immediately after MRI measurements. Tubes were placed on ice, centrifuged and plasma isolated and assayed for triglycerides and cholesterol by using commercially available enzymatic kits from Roche Diagnostics (Mannheim, Germany).

### Statistical analysis

Statistical analyses were performed using GraphPad Prism 5 (GraphPad Software, La Jolla, CA, USA). Values are expressed as mean ± standard error of the mean (SEM). For all statistical comparisons a Mann-Whitney test (unpaired, two-tailed) was applied. For correlation analyses, the Pearson´s correlation coefficient (PCC) was used to assess the validity. Correlation coefficients >0.8 were considered strong, 0.8 to 0.6 substantial, 0.6 to 0.4 moderate, 0.4 to 0.2 fair and 0.2 to 0 almost non-existent, respectively. Probability values p<0.05 were considered as statistically significant.

### Ethic statement

The experiment was supervised by the institutional animal welfare officer and approved by the local licensing authority (Behörde für Soziales, Familie, Gesundheit und Verbraucherschutz; Amt für Gesundheit und Verbraucherschutz, Hamburg, Germany, project no. 33/10).

## Results

### *En face* analyses

**Fig 2:**
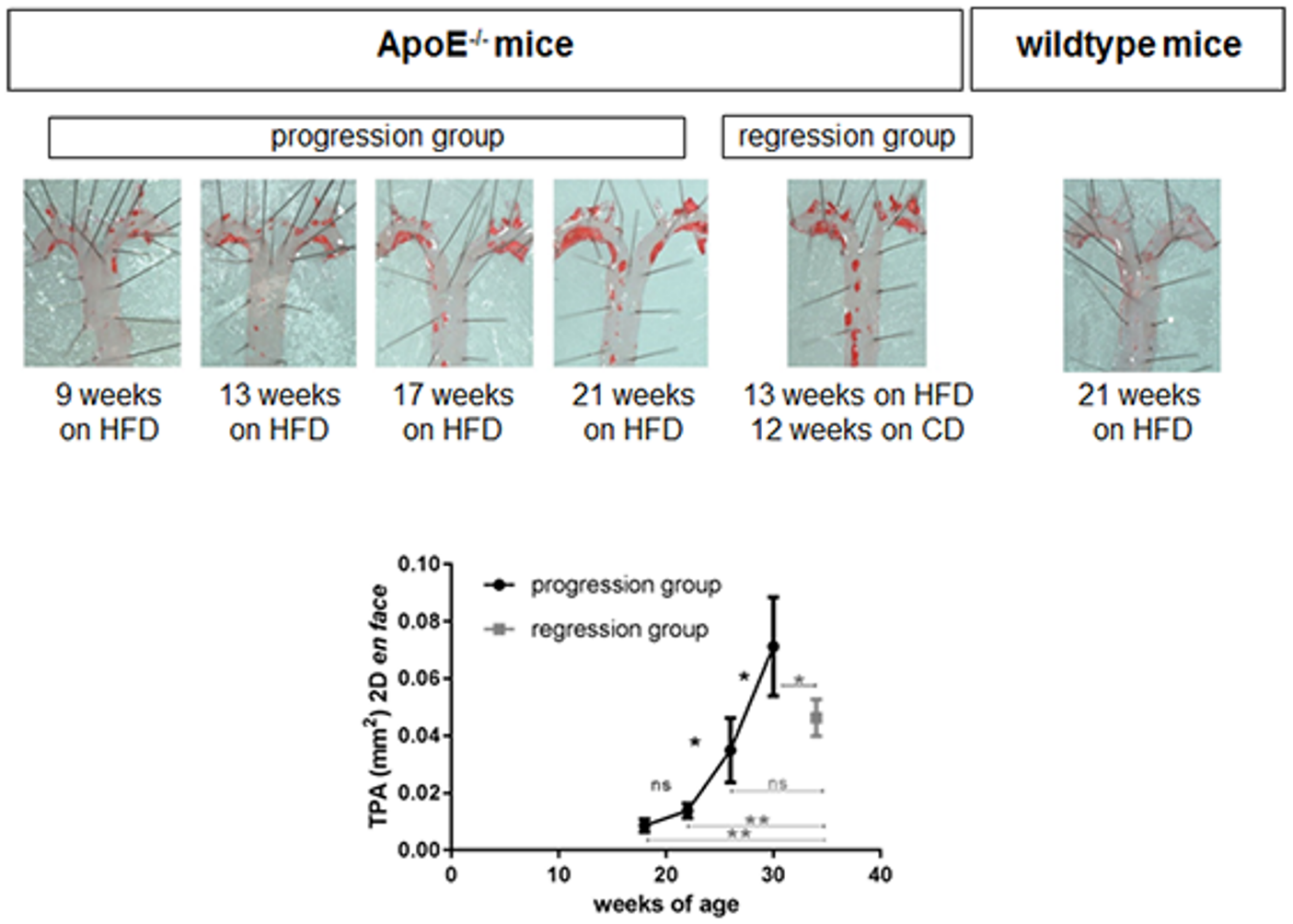
Progression and regression of atherosclerosis adapted from *en face* preparation. *En face* preparation of the aortic arch showing the plaque progression over time. The regression group, which was reswitched to chow diet (CD) 13 weeks after starting high fat diet (HFD) shows a slower increase of total plaque area (TPA). No atherosclerotic lesion was detectable in wildtype mice. (n.s.—non significant, *p<0.05, **p<0.01).

As the Sudan IV staining of the *en face* prepared aortas clearly identifies atherosclerotic lesions, plaque progression over time can be easily visualised and TPA calculated. In Fig 2 one example of each group is represented.

In the progression group (mice remained on HFD), TPA increased exponentially, accelerating from 8.6±2.2×10^−3^ mm^2^ to 13.8±2.4×10^−3^ mm^2^ (p>0.05), 34.9±11.1×10^−3^ mm^2^ (p<0.05) and 71.2±17.3×10^−3^ mm^2^ (p<0.05) at week 9, 13, 17 and 21 (respectively), while in the regression group (mice were switched back to CD after week 13), TPA was 46.3±6.4×10^−3^ mm^2^. However, this value was lower than the TPA of the ApoE^-/-^ mice which remained longest on HFD (71.2±17.3×10^−3^ mm^2^; p<0.05), although these mice were 4 weeks older in age than the others. As expected, no atherosclerotic lesions were detected in wildtype mice.

**Fig 3:**
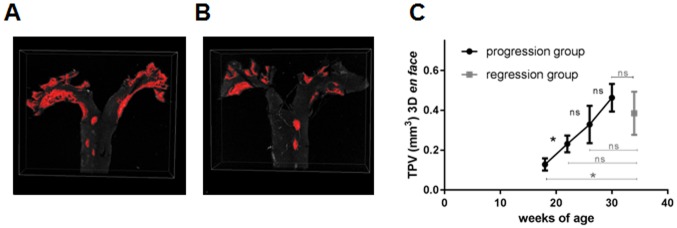
3D plaque volume analysis based on *en face* preparation. **(A)** The total plaque volume (TPV) estimated by 3D confocal microscopy analysis. Shown is an animal of the progression (30 weeks of age) **(A)** and regression group (34 weeks of age) **(B)** with different plaque burden. Plaque progression over time can be estimated by TPV **(C)**. The regression group, which was reswitched to chow diet 13 weeks after starting HFD shows a smaller TPV. (n.s.—non significant, *p<0.05, **p<0.01).

As confocal fluorescence microscopy allows to perform 3D object measurements (Fig 3), TPV of the atherosclerotic lesions was calculated based on the *en face* methods. Due to the fluorescence of the Sudan IV staining plaque areas were clearly identified. As expected, the TPV of the conventional *en face* staining technique displayed an increase of plaque progression over time although the overall increase in plaque size was not that strong. TPV progressed from 0.13±0.03mm^3^ to 0.23±0.04 mm^3^ (p<0.05), 0.33±0.09 mm^3^ (p>0.05) and 0.46±0.07 (p>0.05) mm^3^ (week 9, 13, 17 and 21 of HFD, respectively). However, similar to the TPA, the calculated TPV of the regression group was less in comparison to the ApoE^-/-^ mice, which remained longest on HFD (0.38±0.09 mm^3^; 0.46±0.07mm^3^ respectively; p>0.05).

### MRI analyses

Due to the strong signal enhancement after GDF injection, the plaque burden of atherosclerotic lesions was also estimated *in vivo* over time.

**Fig 4:**
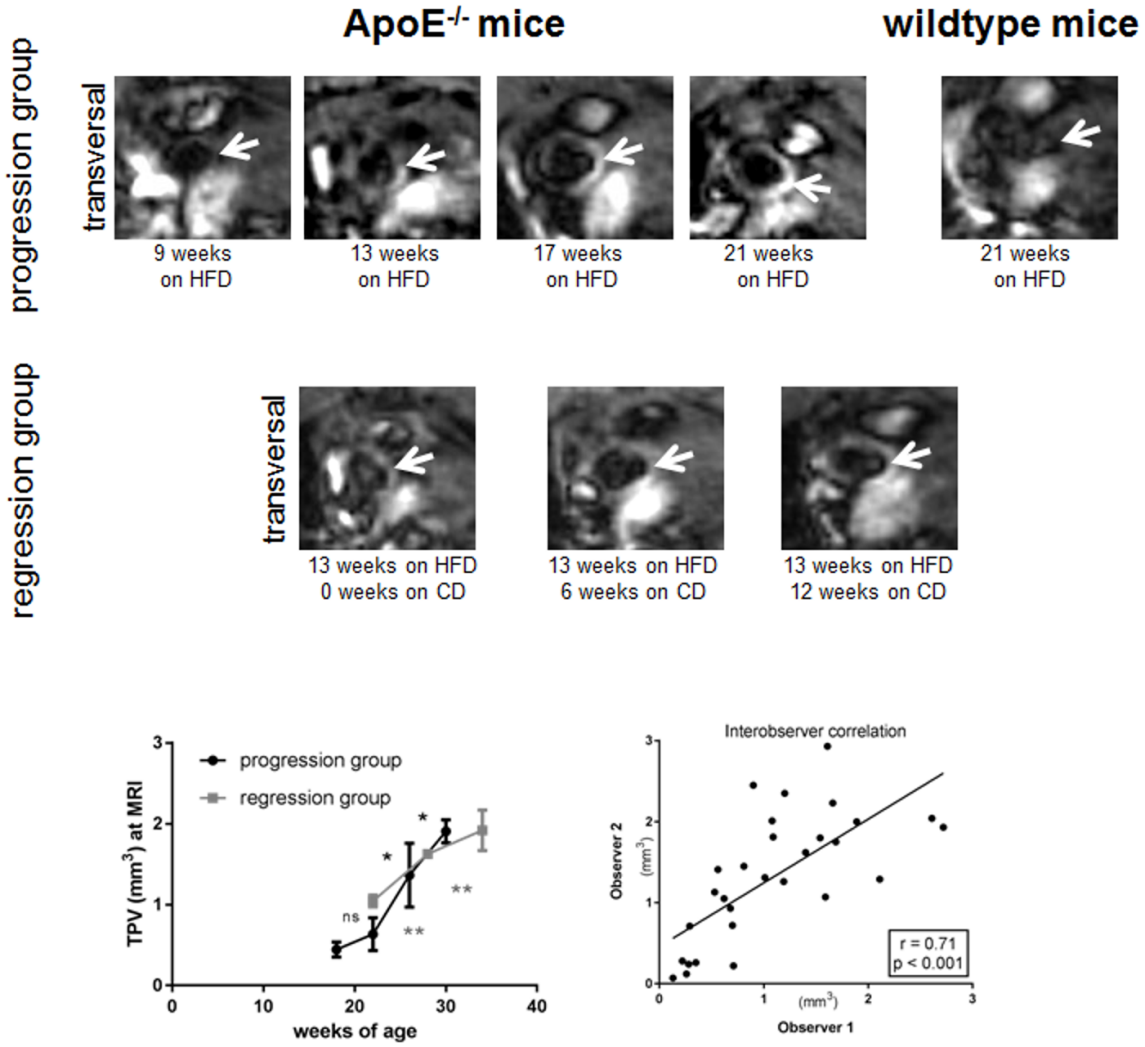
Progression and regression of atherosclerosis at 7T MRI. MRI *in vivo* showed a transversal slice orientation through the ascending aorta. Progression of atherosclerotic lesion (arrow) in ApoE^-/-^ mice can be visualized over time. Calculated TPV showed a more linear increase in case of mouse group reswitching to chow diet (CD) 13 weeks after starting HFD. Interobserver analysis showed a strong and significant correlation of r = 0.71; p<0.001. No plaque was detectable in wildtype mice. (n.s.—non significant, *p<0.05, **p<0.01).

In accordance with the 2D and 3D *en face* analyses the determined TPV by MRI analyses showed a similar exponential increase for the progression group (Fig 4). TPV increased from 0.44±0.09 mm^3^ to 0.63±0.2mm^3^ (p>0.05), 1.36±0.39mm^3^ (p<0.05) and 1.91±0.14mm^3^ (p<0.05; week 9, 13, 17 and 21 of HFD, respectively). Although plaque size increased in the regression group, the progression of plaque burden showed a more linear rise.

Interestingly, the regression group started with a higher plaque burden after being 13 weeks on HFD (TPV of 1.04±0.07 mm^3^) than the ApoE^-/-^ mice in the progression group after 13 weeks on HFD and which remained on HFD (TPV 0.63±0.2 mm^3^).

The interobserver variability demonstrated a substantial and significant correlation for the MR measurements (r = 0.71; p<0.001).

### Correlation analyses

**Fig 5:**
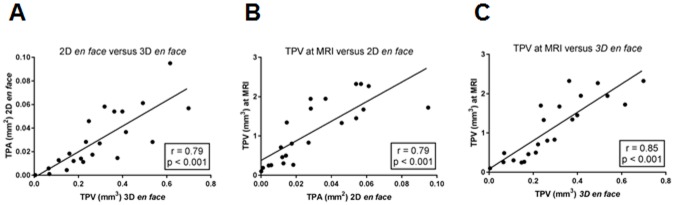
Correlation between plaque size based on MRI and *en face* analysis. Graphs show correlation between total plaque area (TPA) and total plaque volume (TPV) based on *en face* and MRI analysis. Correlation coefficients and p-values are given for 2D *en face* versus 3D *en face* **(A)**, MRI versus 2D *en face* **(B)** and MRI versus 3D *en face* **(C)**. Strongest correlation of r = 0.85; p<0.001 is detectable for the estimated TPV at MRI and 3D *en face*.

To assess the correlation of the plaque areas (TPA) and volumes (TPV) calculated by *en face* analyses and the TPV measured by MRI the Pearson´s correlation coefficient was determined. A strong correlation (r = 0.79; p<0.001) between 2D plaque area and 3D plaque volume based on *en face* methods was observed (Fig 5), and was similar for the correlation between 2D plaque area adapted from *en face* methods and 3D plaque volume based on MRI measurements (r = 0.79; p<0.001). The strongest correlation with r = 0.85 and p<0.001 was achieved between 3D plaque volume adapted from *en face* methods and MRI measurements.

### Quantitative analyses

**Fig 6:**
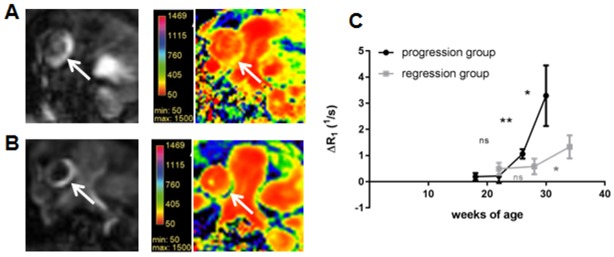
Quantitative MRI analysis using T1 mapping in atherosclerotic plaque. Signal enhancement due to the GDF uptake into the atherosclerotic lesion in one mouse of the progression group (30 weeks of age) **(A)** and in comparison one mouse of the regression group (34 weeks of age) **(B)** with corresponding T1 mapping. In case of progression group T1 relaxation time decreased stronger in comparison to the regression group indicated by the yellow color. The estimated delta R1 revealed a stronger and more exponential acceleration for ApoE^-/-^ mice, which remained on HFD **(C)**. (n.s.—non significant, *p<0.05, **p<0.01).

In agreement with the progression of plaque size over time the estimated ΔR1 based on saturation recovery sequences showed a strong and exponential augmentation for the ApoE^-/-^ mice, which remained on HFD (Fig 6). ΔR1 increased from 0.28±0.1 s^-1^ to 0.5±0.4 s^-1^ (p>0.05), 1.06±0.25 s^-1^ (p<0.01) and 3.29±1.21 s^-1^ (p<0.05; week 9, 13, 17 and 21 of HFD, respectively). The regression group revealed just a slow increase of ΔR1, which increased from 0.49±0.23 s^-1^ to 0.58±0.3 s^-1^ (p>0.05) and 1.33 ± 0.44 s^-1^ (p<0.05; 13, 19 and 25 weeks after switching to ND, respectively).

Pearson´s correlation coefficient was estimated for ΔR1 and plaque size, showing a strong correlation between ΔR1 and TPA (r = 0.79; p<0.001) and a substantial correlation between ΔR1 and TPV (r = 0.69; p<0.01).

### Lipid analysis

**Fig 7:**
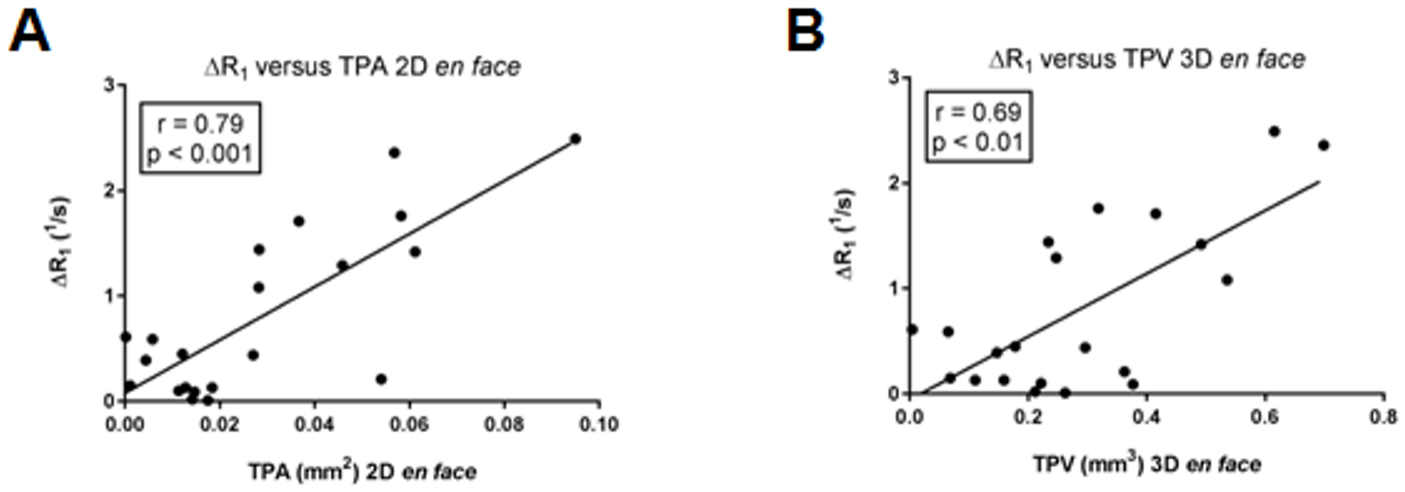
Correlation between T1 relaxivity and plaque size. Graphs show correlation coefficients and p-values for delta R1 versus total plaque area (TPA) **(A)** and delta R1 versus total plaque volume (TPV) **(B)** based on *en face* analysis. Strongest correlation of r = 0.79; p<0.001 is detectable for the estimated delta R1 at MRI and TPA based on 2D *en face*.

To investigate, whether plaque burden and progression correlates with an elevated level of plasma lipids, triglyceride and cholesterol plasma concentrations were estimated. In contrast to plaque size analysis no increase of neither triglyceride nor cholesterol plasma level was detectable for ApoE^-/-^ on HFD until week 21 (Fig 7). Consecutive a negative fair but not significant correlation was found between TPV at MRI and plasma lipids (r = -0.4; p = not significant (n.s.) for cholesterol level and r = -0.3; p = n.s. for triglyceride level).

A moderate but not significant correlation was found for body weight and cholesterol and triglyceride level (r = 0.7; p = n.s. and r = 0.8; p = n.s., respectively).

## Discussion

The study demonstrates, that GDF-enhanced *in vivo* MRI is a powerful non-invasive imaging technique allowing for reliable estimation of plaque burden, monitoring of disease progression and evaluation of therapy response in preclinical studies.

In this study, we report the feasibility to visualize atherosclerotic plaques in ApoE^-/-^ mice at 7T MRI by using GDF, an amphiphilic small molecular weight gadolinium based MRI contrast agent. Changes of plaque development at various stages of atherosclerotic disease were assessed through plaque volume calculation and T1 relaxivity measurements. Both parameters showed an exponential increase over time in case of ApoE^-/-^ mice, which remained on HFD (progression group) and a more linear acceleration in case of the, ApoE^-/-^ mice, which were reswitched to CD after 13 weeks of HFD (regression group). A strong correlation between 2D *en face* analyses and MRI measurements was observed, which was even slightly stronger, if 3D plaque volume adapted from *en face* methods was compared with MRI analysis.

The *en face* preparation of the aortic tree is a popular technique to measure the percentage of lesion area [11,13,15]. We have also demonstrated that the plaque progression and regression can be illustrated by performing *en face* analysis. However, keeping in mind that the *en face* preparation is a time-consuming and technically demanding method, this technique only represents the lesion in a 2D manner with the consequence of an underestimation of the plaque burden. In this study we have demonstrated that confocal microscopy enabled for three dimensional illustrations of atherosclerotic lesions. The application of two-photon microscopy [20] or confocal microscopy for 3D assessment of carotid plaque structure [21] in end-arterectomy specimen and of atherosclerotic plaque in ApoE^-/-^ mouse [22] has been described before but has not been used in *en face* prepared aorta and has not been used to estimate plaque volume. By estimating plaque volume based on the 3D confocal microscopy analysis we were able to reflect the precise volume of the lesion and aorta more accurate than the commonly 2D *en face* analysis.

Nevertheless performing *en face* analysis means that aorta potentially loses its *in vivo* characteristics and may be physically distorted by handling and shrunken by fixation [8].

MRI offers a high contrast in tissue and isotropic voxels for true 3D visualization. Several studies have shown the potential of MRI for plaque detection *ex vivo* [9,23] *as well as in vivo* [24,25]. However previous *in vivo* studies have often shown high resolution in plane (109×109μm), though slice thickness (500μm) had led to disabling partial volume effects [26,27], in which the object of interest, e.g., plaque, did not extend all the way through the imaging slice or is not perpendicular to the plane of the slice. Hockings et al [28] performed 3D MRI *in vivo* in Low-Density Lipoprotein Receptor-Knockout mice using combined cardiac and respiratory triggering resulting in a pixel resolution of 140×187×187 μm and an acquisition time of approximately 30 minutes. However although resolution was superior to resolution in this study (180×180×180μm) acquisition time was threefold longer and voxels were anisotropic. *Ex vivo* studies even achieved voxel size of 47×47×62.5 μm but required approximately seven hours. Moreover, for longitudinal preclinical atherosclerotic studies serial measurements in individual animals *in vivo* were preferable. However we have to point one limitation in this study regarding the comparison of 3D *en face* and MRI volumetry analysis, where we achieved a four-fold-difference. In case of 3D *en face* measurements a threshold intensity was determined in the beginning to use a semiautomatic tool for segmentation and volumetric analysis of atherosclerotic lesions. This higher threshold level leads to smaller plaque volume in comparison to MRI analysis. Nevertheless this study showed that each individual technique can be applied to monitor plaque burden in atherosclerotic disease and a strong correlation was observed despite the difference in plaque volume.

The present study also indicates that T1 mapping of atherosclerotic lesion based on GDF can be used to monitor and quantify progression and inhibition of progression of plaque burden. It is known, that Gadofluorine M, which is similar to GDF, detects increased albumin leakage and deposition of hydrophobic extracellular matrix proteins (including collagens, proteoglycans, tenascin and fibronectin) that are typical for fibroatheromas [15]. As plaque development goes along with enhanced synthesis of extracellular matrix [29], this may explain the exponential increase of ΔR1 in case of progression and the more linear augmentation of ΔR1 in case of regression group. We therefore recommend an inductively plasma mass spectroscopy (ICP-MS) in further studies, to quantify the amount of gadolinium accumulation in the vessel wall and to further correlate the amount with T1 relaxivity measurements. However Phinikaridou et al [30] also showed that relaxation rate (R1) increases with atherosclerotic progression in ApoE^-/-^ mice and decreases in the statin-treated ApoE^-/-^ mice group. In contrast to the presented study MRI examinations were performed using Gadofosveset (Vasovist^®^, Bayer AG, Leverkusen, Germany), which is a gadolinium-based contrast agent that reversibly binds to serum albumin, resulting in a prolonged vascular presence and a 5- to 10-fold increase in relaxivity (r1) [31]. The application of an elastin-specific contrast agent not only allowed to quantify plaque burden in progression and regression group of ApoE^-/-^ mice but also T1 mapping [27].

In this study we used the reswitch to CD 13 weeks after starting the HFD as therapeutic agent as proof of principle to monitor a diminished plaque progression in this group. Due to the knockout of apolipoprotein E in this animal mouse model, mice are very hyperlipidemia and a total regress of plaque burden cannot be expected [32]. However by using GDF enhanced MRI a differentiation between accelerated plaque progression due to HFD and diminished plaque burden due CD is possible. In further preclinical longitudinal studies new therapeutic agents can be evaluated.

### Conclusion

In conclusion, we were able to demonstrate the successful use of GDF-enhanced MRI at 7T for monitoring atherosclerotic plaque progression and regression in a preclinical mouse model. Thus, GDF enhanced MRI represents a promising tool in further preclinical longitudinal atherosclerosis studies to estimate plaque burden and evaluate therapeutic efficacies.
